# Clinical impact of exome sequencing in the setting of a general pediatric ward for hospitalized children with suspected genetic disorders

**DOI:** 10.3389/fgene.2022.1018062

**Published:** 2023-01-09

**Authors:** Maayan Kagan, Rotem Semo-Oz, Yishay Ben Moshe, Danit Atias-Varon, Irit Tirosh, Michal Stern-Zimmer, Aviva Eliyahu, Annick Raas-Rothschild, Maayan Bivas, Omer Shlomovitz, Odelia Chorin, Rachel Rock, Michal Tzadok, Bruria Ben-Zeev, Gali Heimer, Yoav Bolkier, Noah Gruber, Adi Dagan, Bat El Bar Aluma, Itai M. Pessach, Gideon Rechavi, Ortal Barel, Ben Pode-Shakked, Yair Anikster, Asaf Vivante

**Affiliations:** ^1^ Department of Pediatrics B, Edmond and Lily Safra Children’s Hospital, Sheba Medical Center, Tel-Hashomer, Israel; ^2^ Sackler Faculty of Medicine, Tel-Aviv University, Tel-Aviv, Israel; ^3^ Talpiot Medical Leadership Program, Sheba Medical Center, Tel-Hashomer, Israel; ^4^ Pediatric Rheumatology Unit, Edmond and Lily Safra Children’s Hospital, Sheba Medical Center, Tel-Hashomer, Israel; ^5^ Pediatric Nephrology Unit, Edmond and Lily Safra Children’s Hospital, Sheba Medical Center, Tel-Hashomer, Israel; ^6^ The Danek Gertner Institute of Human Genetics, Sheba Medical Center, Tel-Hashomer, Israel; ^7^ The Institute of Rare Diseases, Edmond and Lily Safra Children’s Hospital, Sheba Medical Center, Tel-Hashomer, Israel; ^8^ Pediatric Neurology Unit, Edmond and Lily Safra Children’s Hospital, Sheba Medical Center, Tel-Hashomer, Israel; ^9^ Pediatric Heart Institute, Edmond and Lily Safra Children’s Hospital, Sheba Medical Center, Tel-Hashomer, Israel; ^10^ Pediatric Endocrinology and Diabetes Unit, Edmond and Lily Safra Children’s Hospital, Sheba Medical Center, Tel-Hashomer, Israel; ^11^ Pediatric Pulmonology and National CF Center, Edmond and Lily Safra Children’s Hospital, Sheba Medical Center, Tel-Hashomer, Israel; ^12^ Department of Pediatric Intensive Care, Edmond and Lily Safra Children’s Hospital, Sheba Medical Center, Tel-Hashomer, Israel; ^13^ The Wohl Institute for Translational Medicine, Sheba Medical Center, Tel-Hashomer, Israel; ^14^ Sheba Cancer Research Center, Sheba Medical Center, Tel-Hashomer, Israel; ^15^ The Genomics Unit, Sheba Cancer Research Center, Sheba Medical Center, Tel-Hashomer, Israel; ^16^ Metabolic Disease Unit, Edmond and Lily Safra Children’s Hospital, Sheba Medical Center, Tel-Hashomer, Israel

**Keywords:** exome sequencing (ES), general pediatrics, monogenic, hospitalized, inpatient

## Abstract

**Background:** Genetic conditions contribute a significant portion of disease etiologies in children admitted to general pediatric wards worldwide. While exome sequencing (ES) has improved clinical diagnosis and management over a variety of pediatric subspecialties, it is not yet routinely used by general pediatric hospitalists. We aim to investigate the impact of exome sequencing in sequencing-naive children suspected of having monogenic disorders while receiving inpatient care.

**Methods:** We prospectively employed exome sequencing in children admitted to the general pediatric inpatient service at a large tertiary medical center in Israel. Genetic analysis was triggered by general and/or subspecialist pediatricians who were part of the primary inpatient team. We determined the diagnostic yield among children who were referred for exome sequencing and observed the effects of genetic diagnosis on medical care.

**Results:** A total of fifty probands were evaluated and exome sequenced during the study period. The most common phenotypes included were neurodevelopmental (56%), gastrointestinal (34%), and congenital cardiac anomalies (24%). A molecular diagnosis was reached in 38% of patients. Among seven patients (37%), the molecular genetic diagnosis influenced subsequent clinical management already during admission or shortly following discharge.

**Conclusion:** We identified a significant fraction of genetic etiologies among undiagnosed children admitted to the general pediatric ward. Our results support that early application of exome sequencing may be maximized by pediatric hospitalists’ high index of suspicion for an underlying genetic etiology, prompting an in-house genetic evaluation. This framework should include a multidisciplinary co-management approach of the primary care team working alongside with subspecialties, geneticists and bioinformaticians.

## 1 Introduction

Genetic etiologies are responsible for a substantial portion of pediatric diseases. Yet, in many cases genetic diagnosis is hindered or completely missed (Schroeder et al., 2021). In the past decade, owing to advancements in the field of genetics and the growing accessibility of next generation sequencing (NGS) methods, exome sequencing (ES) has become a powerful tool, shedding light on many genetic cases that were previously under-detected (Boycott et al., 2013; Yang et al., 2013).

Nowadays, the use of ES is a key for the identification of monogenic disorders which often cannot be clinically diagnosed. ES can identify single gene diseases (also called Mendelian or monogenic diseases). Thus, it encompasses a substantial portion of genetic alternations providing accurate and timely molecular diagnoses across a myriad of pediatric conditions (Singleton, 2011; Xue et al., 2015; Retterer et al., 2016).

In recent years, ES has been used more prevalently in the research as well as clinical settings, as it became more available and less costly. From a clinical perspective, it is prominently used in the setting of outpatient genetic consultations or clinics (Pode-Shakked et al., 2021). Nonetheless, recent implications of ES encompass diagnosis and management of patients in different pediatric subspecialties across a multitude of disease etiologies (Dixon-Salazar et al., 2012; Lee et al., 2014; Zhang et al., 2015; Pode-Shakked et al., 2021; Pode-Shakked et al., 2022). Early implementation of ES can shorten the lengthy odyssey of the diagnostic process, shorten hospitalizations, spare unnecessary invasive procedures, reduce medical expenses, and may alter surveillance and management at early stages, which could be crucial in certain cases (Valencia et al., 2015; Splinter et al., 2018; Wise et al., 2019; Manickam et al., 2021). Still, ES is rarely performed in the setting of general pediatric wards for hospitalized children. Consequently, data regarding its diagnostic yield, benefits and challenges during acute admission in this patient population is scarce.

Herein, we present a single center experience employing ES as part of the pediatric in-patient service for patients suspected by the pediatric hospitalist team to have an underlying and previously undiagnosed genetic condition.

The rationale behind the decision to routinely establish ES as an available medical test to the general pediatric ward team, was derived from the accumulating knowledge on the molecular basis of pediatric diseases, the growing availability of NGS techniques, and our previous experience with undiagnosed medical cases from the past (pre-ES era). In this respect, we recently sequenced multiple cases of unsolved medical mysteries from the past and from whom we had available DNA samples (Table 1). These cases highlighted the diagnostic power of ES and the need for quick and accurate molecular diagnosis. Subsequently, we initiated a designated hospitalists team working in collaboration with medical geneticists and subspecialist consultants, in order to provide a ‘one-stop-shop,’ in which patients suspected to harbor an underlying genetic etiology during their hospitalization, would undergo a timely and multifaceted genetic evaluation and analysis. Indeed, the aim of this initiative was never to transfer the routine care of these patients affected with different genetic diseases from their primary provider/geneticist, but rather to promptly initiate genetic evaluation and shorten the diagnostic odyssey for such families, based on an in-house team of experts well familiar with the genetic landscape of different pediatric disorders.

**TABLE 1:** Characteristics of a historical cohort of undiagnosed children which were solved following exome sequencing.

Group	Patient	Age (years)	Parental consanguinity		Gender	Phenotype	Gene (variant)/CNV	Zygosity	Diagnosis (OMIM)
*Cardiovascular*									
	A	9		No	F	Mitral valve prolapse, scoliosis	***FBN1*** c.1097G>A, p.Trp366^a^	Heterozygous	Marfan syndrome (154700)
	B	0.5		No	M	Cardiomyopathy, hypoglycemia	***MYH7*** c.2775G>C, p.Arg925Ser	Heterozygous	Myopathy, myosin storage, autosomal dominant (160760)
*Central Nervous System*									
	C	3		No	M	Epilepsy, developmental delay, hypoglycemia	16p del (11.2-12.2)	Heterozygous	Chromosome 16p11.2 deletion syndrome (611913)
	D	7		Yes	M	Developmental delay, chorioretinitis, brain atrophy	***POLR3A*** c.950G>A, p.Cys317Tyr	Homozygous	Wiedemann-Rautenstrauch syndrome (614258)
*Multi-systemic*									
	E^a^	0		Yes	F	Dysmorphism, TOF, CKD	***TMEM260*** c.1698_1701delTC, p.Tyr567Thrfs^a^27	Homozygous	Structural heart defects and renal anomalies syndrome (617478)
	F	0		Yes	M	Developmental delay, cataract, deafness, microcephaly	***RAB3GAP1 *** 80delIT, Ile27fs	Homozygous	Warburg Micro syndrome-1 (600118)
	G	1		Yes	F	Developmental delay, elevated liver enzymes, FTT	***ERCC6*** c.2870T>G, p.Val957Gly	Homozygous	Cockayne (609413)
*Rheumatology*									
	H^a^	3		Yes	F	Enlarged MCP and PIP	***CCN6*** c.257G>T p.Cys86Phe	Homozygous	Progressive pseudorheumatoid arthropathy of childhood (208230)
*Metabolic*									
	I	0.1		Yes	F	Hypoglycemia	***ABCC8*** c.946G>A, p.Gly316Arg	Homozygous	Hyperinsulinemic hypoglycemia, familial, 1 (600509)
	J	9		No	M	Hepatomegaly, elevated liver enzymes	***PHKA2*** c.3603_3606dupTGAC, p.Ser1203Ter	Hemizygous	Glyogen storage disease IXA (300798)
	K	0		No	F	Hyperammonemia, elevated citrulline blood level	***ASS1*** c.535T>C, p.Trp179Arg	Homozygous	Citrullinemia (215700)

Abbreviations: CKD, chronic kidney disease; CNV, copy number variation; FTT, failure to thrive; MCP, metacarpophalangeal; NA, not available; PIP, proximal interphalangeal; TOF, tetralogy of Fallot;

^a^ These cases were previously published (Ta-Shma et al., 2017; Pode-Shakked et al., 2019).

## 2 Materials and methods

### 2.1 Patients’ characteristics and the general pediatric ward

The Edmond and Lily Safra Children’s Hospital at the Sheba Medical Center (SMC) is a tertiary center accepting acute pediatric patients as well as chronic and/or complicated cases from all across the country. In addition, patients are admitted from the Palestinian National Authority (∼20% of the hospitalized children) as well as complex cases from different parts of the world. The SMC General Pediatric Ward is specialized in offering expert care to children from birth to late adolescence. The General Pediatric Ward team, with the aid of the hospital’s consulting services, is highly experienced in treating children with acute and chronic illness, complex medical conditions and syndromes, inborn errors of metabolism, diverse types of cancer, congenital anomalies and others. Children are typically admitted for ∼4 days on average during which diagnostic evaluations and treatment plans are executed.

### 2.2 Prospective patient selection for genetic analysis during acute hospital admission

All patients were admitted to the general pediatric ward at the Edmond and Lily Safra Children’s Hospital at SMC, Israel, between the years 2019–2022. A designated physician from the in-patient team was appointed as the genetics coordinator of the ward. Patients suspected to harbor a genetic etiology were presented daily during medical rounds to the coordinator. Subsequently, the coordinator offered each relevant family the option of genetic testing free of charge. Children for whom a specific diagnosis was highly suspected, and only a single or few genes (or mutations) were tested, were not included in this study. Each family received a detailed explanation on ES analysis, along with the advantages and pitfalls of the technique, with special attention given to secondary and incidental findings. Subsequently, a written informed consent was obtained from each interested family. The coordinator gathered probands’ and their families’ medical history and drew an annotated pedigree. Subsequently, blood samples were obtained from the proband and when feasible from additional family members, and were used for DNA extraction.

For coverage of ES costs, we used a designated fund of the children’s hospital supported by SMC. Consequently, for patients who required genetic testing, we were able to provide ES free of charge. The study was conducted in accordance with the tenets of the Declaration of Helsinki, and under approval of the Institutional Review Board at the SMC.

### 2.3 Clinical evaluation

Baseline characteristics were recorded for each patient upon enrollment, including current age, gender, age and phenotype at presentation, ethnic origin and parental consanguinity. The clinical symptoms were presented as HPO terms to the bioinformatics laboratory based on the clinical information. In addition, we completed a scan of the electronic medical chart for additional characteristics, such as primary clinical diagnosis and medical workup that was completed prior to the genetic testing (i.e., blood tests, imaging, etc).

### 2.4 Exome sequencing and bioinformatic analysis

We performed exome sequencing, variant detection, and filtering as previously described (Tirosh et al., 2019). Variant calling was performed by a team of clinician scientist bioinformaticians, who had knowledge of the clinical phenotypes and pedigree structure, as well as genetic expertise in exome evaluation as previously described (Bolkier et al., 2021). Exome data were interpreted according to the American College of Genetic and Genomic Medicine (ACMG) guidelines. During the ES initial evaluation and thereafter, the pediatrics hospitalist team was in close discussion with the in-house SMC bioinformatician team regarding the detected variants as well as filtering process. Patients who were found to harbor a variant classified as pathogenic or likely pathogenic were defined as having a positive molecular diagnosis. Patients suspected to harbor a genetic etiology and required further investigation, such as segregation analysis, were defined as having a non-conclusive ES result.

### 2.5 Genetic results reporting and follow up

Following ES results, families were invited for a follow-up visit with geneticists/primary provider, during which recommendations and changes to clinical management were recorded and further referral for additional consults or diagnostic tests was done. Additionally, explanations regarding risk of recurrence in future pregnancies and family planning options were conveyed.

## 3 Results

### 3.1 Patients and phenotypes

#### 3.1.1 Historical cohort

Table 1 shows a historical cohort of eleven undiagnosed children admitted to our ward in the pre-ES era, which were solved following ES. These cases included children with ultra-rare medical conditions or children exhibiting non-specific phenotypes hampering the establishment of a single or accurate genetic diagnosis.

#### 3.1.2 Prospective cohort

Baseline characteristics of fifty patients recruited prospectively to the study are summarized in Table 2. Patients were predominantly male and 22% were offspring of consanguineous unions. Single/duo ES was performed in 68% and trio ES was performed in 32% of cases. The most common phenotype in our cohort involved the central nervous system (CNS) (56%), gastrointestinal system (34%), and cardiovascular system (24%). In 32% dysmorphic features were noted. ES revealed a genetic diagnosis in 38% of the cohort (Table 3). Four patients (8%) had non-conclusive ES results which require further investigation (Supplementary Table S1). Notably, patients with a positive ES result had higher rates of CNS involvement compared to other phenotypes (∼63%), followed by gastrointestinal (∼42%) and facial dysmorphism (∼42%) phenotypes. Of the solved cases, three had a *de novo* variant detected by trio-analysis. Five out of the nineteen solved cases were from consanguineous families.

**TABLE 2:** Demographic and phenotypic characteristics of the fifty probands evaluated by exome sequencing (ES).

Characteristics	Value no (%)
Gender	
Female	21 (42.0%)
Male	29 (58.0%)
Age (years)	
Mean	5.4
Range	.2–17.8
Ethnicity	
Ashkenazi Jews	10 (20.0%)
Sephardic Jews	5 (10.0%)
Mixed Ashkenazi-Sephardic ethnicity	10 (20.0%)
Arab	17 (34.0%)
Kurdish	2 (4.0%)
Others	6 (12.0%)
Consanguinity	11 (22.0%)
Type of ES	
Single	33 (66.0%)
Duo	1 (2.0%)
Trio	16 (32.0%)
Primary phenotype at presentation^a^	
Central nervous system	28 (56.0%)
Gastrointestinal (including failure to thrive)	17 (34.0%)
Facial dysmorphism	16 (32.0%)
Cardiovascular	12 (24.0%)
Musculoskeletal (bone, connective tissue and muscle)	11 (22.0%)
Nephrology/Urology	10 (20.0%)
Oncology	6 (12.0%)
Hematology and coagulation	6 (12.0%)
Rheumatology	6 (12.0%)
Endocrine	6 (12.0%)
Metabolic	5 (10.0%)
Neurovascular	4 (8.0%)
Ophthalmology	3 (6.0%)
Respiratory	2 (4.0%)
Immunology	2 (4.0%)
Otolaryngology	2 (4.0%)
Neuromuscular	2 (4.0%)
ES result	
Positive	19 (38.0%)
Negative	27 (54.0%)
Non-conclusive	4 (8.0%)
Total	50

^a^ Each patient may present with more than one phenotype.

**TABLE 3:** Patients for whom genetic workup yielded a molecular diagnosis, shown according to clinical diagnosis and phenotype.

Group	Patient	Age (years)	Consanguinity	Gender	Phenotype	Type of ES	Gene (variant)/CNV	ACMG	Zygosity	OMIM
*Cardiovascular*										
	**1**	1.3	Yes	M	AV canal, FTT, dysmorphism, low set ears, high arch palate, inguinal hernia, hypotonia, polydactyly (hands), syndactyly (feet), nail hypoplasia	Single	*EVC* (c.612C>A; p.Cys204ter)NM_153717.3	*PVS1, PM2, PP5, Clinvar ID: RCV001222961*	Homozygous	Ellis-van Creveld syndrome (225500)
	**2**	1.8	No	F	Dilated cardiomyopathy, thrombocytopenia	Trio	*TNNI3* (c.258del; p.Leu88TrpfsTer27) NM_000363.5	*PP5, PVS1, PM2, PP1, Clinvar ID:* RCV001333390	Homozygous	Cardiomyopathy, dilated (191044)
	**3**	11.7	No	F	Supravalvular aortic stenosis, peripheral pulmonary artery stenosis, tall stature, mild scoliosis	Single	*ELN* (c.591delA; p.Pro198ArgfsTer111) NM_001278916.2	*PVS1, PM2*	Heterozygous	Williams-Beuren syndrome (194050)
*Central Nervous System*										
	**4**	0.5	No	M	Epilepsy, WPW, developmental delay, autism, macrocephaly, spinal lipoma	Trio	*PTEN* (c.1271G>A; p.Gly424Asp) NM_000314.8	*PM1,PP2,PM2, PM5,PP3, PP5, Clinvar ID: RCV001762656*	Heterozygous (maternally inherited)	Cowden syndrome (158350)
	**5**	0.6	No	F	Developmental delay, neonatal hypotonia, FTT	Trio	*GRIN2B* (c.1661T>G; p.Phen554Cy) NM_000834.5	*PM2, PM1, PP2, PS2*	Heterozygous (DN)	Developmental and epileptic encephalopathy (138252)
*Gastrointestinal*										
	**6**	0.3	No	M	FTT, dysmorphism, hypotonia, neonatal apnea, chronic diarrhea	Single	*Del 7.7 Mb*: Chr8: 240885-7977995*, Dup 20.8 Mb*:Chr8: 12737984-33551095; hg19	PMID: 34282301	Heterozygous	NA
*Multi- systemic*										
	**7** ^a^	0.3	Yes	F	Cardiomyopathy, cataract, absent corpus callosum, hypotonia, global developmental delay	Single	*EPG5* (c.1461delC; p.Ala488LeufsTer32) NM_020964.3	*PVS1,PM2*	Homozygous	Vici syndrome (242840)
	**8**	0.4	No	M	Recurrent apnea, dysplastic corpus callosum, sinus vein thrombosis, hypercalcemia, hypospadias, microretrognathia	Trio	*USP7* (c.1175G>C: p.Gly392Ala) NM_003470	*PM2, PP2, PP5, Clinvar ID: RCV001788549*	Heterozygous (DN)	Hao-Fountain syndrome (616863)
	**9** ^a^	1	No	F	Pancytopenia, RTA, FTT, seizures	Trio	*Del 6713bp:* m.9101-m.15816, 65% heteroplasmy	NA	NA	Pearson marrow-pancreas syndrome (557000)
	**10**	4.8	No	F	PKU, nephrotic syndrome	Trio	*PAH* (c.728G>A p.Arg243Gln; c.1241A>G p.Tyr414Cys) NM_001354304.2	c.728G>A p.Arg243Gln- PM1 PP2 PM2 PM5 PP3, Clinvar ID: PP5 RCV000000622; c.1241A>G- PS3 PM1 PP2 PM2 PM5 PP3 PP5, Clinvar ID: RCV000150074	Compound heterozygous	Phenylketonuria (261600)
	**11**	2.7	No	F	Short stature, short neck, developmental delay, long philtrum, synophris, hypertrichosis, intention tremor	Trio	*PUF60* (c.T245_246delTG; p.Val81del) NM_001271096	*PVS1, PM2*	Heterozygous	NA
	**12**	16.5	No	F	Psychiatric disorder, intellectual disability, morbid obesity, diabetes, acanthosis nigricans, splenomegaly, scoliosis, brachydactyly, short stature	Single	*Del 1.8 Mb*: Chr17: 16939537-18778764- Hg38	PMID: 21897445	Heterozygous	Smith-Magenis syndrome (607642)
*Oncology*										
	**13**	7.9	No	F	CNS lesion, multiple demyelination lesions, Café au lait	Trio	*MSH6* (c.1444C>G p.Arg482Gly) NM_000179.3, (c.3984_3987dup chr2-47806630 A>ATCAG	*c.1444C>G p.Arg482Gly PM2 PM5 PP3 PP5, Clinvar ID: RCV001267895 MSH6:c.3984_3987dup PVS1 PM2, Clinvar ID: PP5RCV000074964*	Compound heterozygous	Mismatch repair cancer syndrome (619097) p.Leu1330fs) NM_000179.3
	**14**	2.5	Yes	F	Juvenile myelomonocytic leukemia	Single	*NRAS* (c.37G>C; p.Gly13Arg) NM_002524.5	*PM1 PP2 PM2 PM5 PP3 PP5*	Heterozygous	Noonan syndrome (613224)
	**15**	1	Yes	F	Ophthalmic lesion, spinal lesion, Café au lait, hypomelanotic macule	Single	*NF2* (c.784C>T; p.Arg262*) NM_000268.4	*PVS1 PM2 PP5, Clinvar ID: RCV000003451*	Heterozygous	Neurofibromatosis, type 2 (101000)
	**16**	0.8	No	M	Hirsutism, abnormal endocrine laboratory tests, renal cysts	Trio	*TP53* (c.742C>T; p.Arg248Trp) NM_000546.6	*PP1 PS3 PS4 PS1, Clinvar ID: RCV00016824*	Heterozygous	Li-Fraumeni syndrome (151623)
*Rheumatology*										
	**17**	13.6	No	M	Skin lesions, rash, Raynaud’s syndrome, kyphosis, lordosis, decreased joints range of motion	Trio	*LMNA* (c.139G>T p.Asp47Tyr) NM_170707.4	*PM1 PP2 PM2 PP3 PS2*	Heterozygous (DN)	Familial Partial Lipodystrophy, Dunnigan type (150330)
	**18** ^a^	9.1	Yes	F	Multiple bone lesions suspected for CRMO, s/p medulloblastoma, hyperphosphatemia, dental abnormalities	Single	*GALNT3* (c.1524 + 1G>A) NM_004482.4	*PVS1 PM2 PP5, Clinvar ID: RCV000008234*	Homozygous	Tumoral calcinosis, hyperphosphatemic, familial (211900)
*Other*										
	**19**	2.7	No	M	Multiple subcutaneous bony lesions	Single	*ACVR1* (c.617G>A; p.Arg206His) NM_001111067.4	*PP3 PM2 PP5*	Heterozygote	Fibrodysplasia ossificans progressiva (135100)

Abbreviations: AV, atrioventricular; CNS, central nervous system; CRMO, chronic recurrent multifocal osteomyelitis; CVS, copy number variation; DN, *de novo*; ES, exome sequencing; FTT, failure to thrive; F, female; M, male; PKU, phenylketonuria; RTA, renal tubular acidosis; WPW, wolf parkinson white.

^a^ These cases have been previously published (Chorin et al., 2022; Pode-Shakked et al., 2022).

### 3.2 Effect of diagnosis on medical care

**FIGURE 1:**
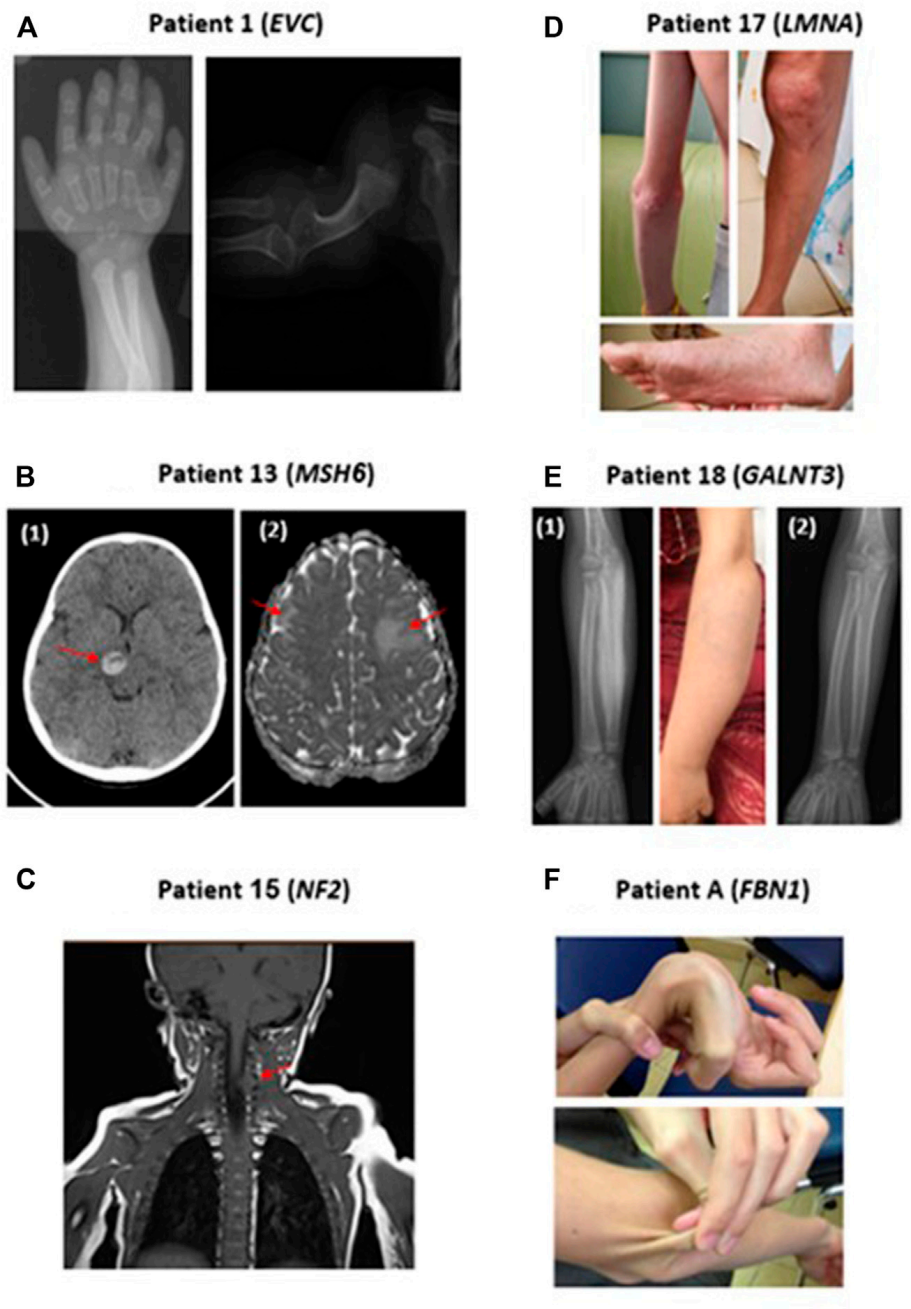
Representative clinical findings in patients with positive molecular diagnosis in our cohort. **(A)** *Patient 1*: Radiographs demonstrating polydactyly and deformation of the humerus, in a patient with Ellis-Van Creveld syndrome (*EVC* mutation). **(B)** *Patient 13*: Brain CT (1) and MRI (2) scans demonstrating hemorrhage of the thalamus and bilateral frontal lesions in a patient with compound heterozygous *MSH6* mutations. **(C)** *Patient 15*: MRI scan preformed due to orbital lesion detected an incidental finding of a spinal lesion at C4-5 level in a patient with *NF2* mutation. **(D)** *Patient 17*: Patient with *LMNA* mutation presenting with widespread telangiectasias and Scleroderma-like skin changes. **(E)** *Patient 18*: (1): Hyperostosis of the right ulna secondary to *GALNT3* mutation demonstrated in radiographs and clearly visible in the adjacent photograph (2) Radiographs of the right Ulna following phosphorus chelator therapy, demonstrating a substantial improvement of hyperostosis. **(F)** *Patient A:* Patient with Marfan syndrome presenting with joint hypermobility and hyper elastic skin.

The timely genetic diagnoses affected medical care and allowed a personalized approach in a variety of ways. The establishment of accurate and correct root cause in a short period of time prevented treatment delays as well as unnecessary interventions or diagnostic procedures. Moreover, in six children (∼32%) ES opened a window for optional personalized therapy, and in several children (∼37%), ES results had a direct effect on medical management during admission or shortly following discharge as highlighted below in three representative cases:

Patient 18 (Table 3) presented with 4 months of worsening arm swelling and suspected chronic recurrent osteomyelitis (CRMO) (Figure 1). Her past medical history was notable for medulloblastoma at the age of 2 years. Prior to her hospitalization, she had an extensive diagnostic work up at the oncology clinic. MRI and PET-CT of the arm were suspected for Ewing sarcoma. Open bone biopsy ruled out malignancy and was consistent with callus formation and associated mild chronic inflammation. During her hospitalization, we noted hyperphosphatemia with normal kidney function which raised the possibility of abnormal phosphate regulation. Additionally, the fact that her parents were first degree cousins, supported the possibility of a monogenic recessive disorder. ES was completed and treatment of phosphate chelators was initiated. A homozygous *GALNT3* variant which was previously reported to cause Hyperostosis Hyperphosphatemia Syndrome was detected (Topaz et al., 2004).

Elucidating the molecular diagnosis, in this case, prevented treatment with bisphosphonates for CRMO which could have worsened her condition. Subsequently, the patient’s two siblings were tested for plasma phosphate level and found to have hyperphosphatemia. In summary, this case highlights the importance of a high index of suspicion for genetic etiologies in children with parental consanguinity. Radiographs of this patient’s arm before and after treatment are presented in Figure 1.

Patient 13 (Table 3) presented with 2 weeks of headache followed by acute weakness of her left arm and leg. On examination she had hemiparesis, as well as multiple café au lait spots. Brain CT and MRI showed bleeding of her right thalamus and bilateral frontal lesions (Figure 1). Since these findings were radiologically suspected for acute disseminated encephalomyelitis (ADEM), the patient was treated with high dose intravenous methylprednisolone. The combination of brain lesions and café au lait spots, led to consideration of an underlying genetic etiology secondary to mutations in *NF1* or genes coding to mismatch repair proteins such as *MSH2* or *MSH6*. Rapid ES exam results were notable for compound heterozygous variants in *MSH6*, a mismatch repair gene. Pathogenic variants in this gene are known for increasing the risk of colorectal and CNS malignancies. Consequently, the patient was followed by the oncology and neurosurgery teams. Subsequent brain biopsy was performed and showed grade II astrocytoma. Segregation analysis showed that each of her parents was a carrier for one of the two variants, and therefore are also at increased risk for malignancies (Lynch syndrome), requiring additional medical follow-up. In addition, the parents decided to undergo preimplantation genetic diagnosis (PGD) for future pregnancies. This case illustrates how early suspicion and employment of ES not only changed the diagnosis and management of the patient, from a suspected autoimmune condition to malignancy, but also influenced the child’s immediate family in terms of informed anticipatory follow-up and family planning.

Patient 17 (Table 3) was admitted for evaluation of low body mass index (below first centile), Raynaud’s phenomenon, worsening sclerodermic-like skin lesions, telangiectasia and joint contractures (Figure 1). The initial differential diagnosis included autoimmune connective tissue disease versus genetic causes of lipodystrophy. A comprehensive diagnostic work-up, including gastroenterology and rheumatology testing were completed. ES revealed the rare diagnosis of heterozygous *LMNA* mutation causing Familial Lipodystrophy type 2 (Dunnigan type), a previously reported cause for scleroderma-like presentation (Akinci et al., 2000). In this case, establishing a molecular genetic diagnosis allowed an unequivocal disease diagnosis and opened a window for tailored medical treatment and monitoring, as well as for possible novel treatment options such as apolipoprotein C III inhibitors (Aslesh and Yokota, 2020; Lightbourne et al., 2021).

## 4 Discussion

In this study, we present data on the diagnostic yield of next-generation sequencing and its effect on medical care and follow-up, among sequencing-naive children suspected of having a monogenic disorder while receiving acute or chronic inpatient care at the general pediatric department. We found that ES during acute hospitalizations significantly contributed to personalized management. Moreover, we show that even though many patients had long standing conditions, only following the genetic analysis which was initiated during their hospitalization, was a molecular diagnosis reached. This was very much due to the dedication and high index of suspicion of the primary hospitalists team to pursue timely and efficient genetic analysis, and with no costs to the families, thus eliminating diagnostic disparities based on financial resources (Grant et al., 2021). The limitations of our study include a relatively small and selective cohort, a single-center based cohort with a relatively high consanguinity rate (22%), uncertainty regarding *de novo* mutations since in most cases we performed a proband-only (single) exome, and the lack of complimentary data obtained by chromosomal microarray for detection of copy number variations. Additionally, a designated fund enabled us to offer ES to all recruited patients at no cost to the families, eliminating disparities based on their insurance coverage or financial constraints, however we recognize that this might not be feasible in every medical center or healthcare system.

Our study highlights several important conclusions for the diagnosis of monogenic disease in the setting of the general pediatric ward. First, as mentioned above, the patient population consists of infants and children hospitalized at a general pediatric ward, rather than critically ill patients receiving intensive care. This is noteworthy as the relatively high diagnostic yield demonstrates the significant portion of genetic etiologies not only among severely affected children requiring complex care, but also those manifesting with more common general pediatric diseases. As a result, pediatric hospitalists may be the first clinicians to be approached, highlighting the importance of a high index of suspicion to the possibility of genetic etiology. Second, our results reflect the real-world clinical utility of ES, in contrast to research-based cohorts, which are more often those reported in the literature, especially for children with neurodevelopmental disorders.

While the diagnostic yield and medical implications of clinical ES in neonatal and pediatric intensive care units have been widely studied (Meng et al., 2017; Freed et al., 2020; Lunke et al., 2020; Liu et al., 2021; Ouyang et al., 2021; Scholz et al., 2021), its clinical utilization in the in-patient setting of a general pediatric ward has scarcely been reported. We identified a monogenic etiology in 38% of children suspected to have undiagnosed genetic conditions. Importantly, a significant portion of the cases we identified were related to lifelong cancer risk (e.g., *TP53, NF2, NRAS, PTEN*, and *MSH6*), affecting both informed anticipatory follow-up and anticancer treatment regimens for these children and their families (Zhang et al., 2015; Scollon et al., 2021). Of note, prior to every ES test, a formal explanation and conversation was made with the families during which the team has explained the possibility of secondary/incidental findings, as well as possible findings which may confer future cancer risk.

As the field of medical genetics continues to advance and becomes more accessible, it is growingly recognized that physicians at every stage of their career should become familiarized with the basis of genetics, which will provide them with the tools to diagnose patients with genetic diseases. Hence, we believe that proper genetic education is a steppingstone and essential at all training levels. Nonetheless, as genetics become an inherent part of in-patient care, medical genetics health providers, physicians and genetic counselors should be an integral part of the ward to provide in depth analysis within the ward, complete auxiliary testing including segregation analysis following initial results and offer novel treatments. Furthermore, following familial analysis, family planning tools and parental counselling are crucial.

A molecular genetic diagnosis can shorten the diagnostic process, prompt targeted therapy, spare unnecessary interventions and diagnostic procedures, and has the potential to lower the costs of the diagnostic process and prevent birth of additional affected family members (Lavelle et al., 2022).

In summary, we report herein the experience of using NGS tools in a pediatric ward of a tertiary hospital for rapid and accurate diagnosis of complex diseases. We identified a significant fraction of genetic etiologies among previously undiagnosed children admitted to the general pediatric ward, which impacted subsequent clinical management. The pediatric hospitalists’ high index of suspicion for an underlying genetic etiology prompted in-house genetic evaluation. Our experience portrays the important need to integrate genetics in hospital work of general pediatric hospitalists and pediatric subspecialties, requiring the current in-house pediatric hospitalist to have in-depth knowledge of genetics, along with close collaboration with bioinformaticians and geneticists allowing provision of personalized care.

## Data Availability

The original contributions presented in the study are included in the article/Supplementary Materials, further inquiries can be directed to the corresponding author.
