# Supplementary material for: Clinical impact of exome sequencing in the setting of a general pediatric ward for hospitalized children with suspected genetic disorders
Source: Front Genet. 2023 Jan 9;13:1018062. doi: 10.3389/fgene.2022.1018062 (PMC9868164; doi:10.3389/fgene.2022.1018062)
Supplement: Supplementary file 1 [file Table1.DOCX]

| **Supplemental Table 1: Patients for whom genetic workup yielded inconclusive results requiring further investigations** | | | | | | | |  |
| --- | --- | --- | --- | --- | --- | --- | --- | --- |
|  |  |  |  |  |  |  |  |  |
| **OMIM** | **Zygosity** | **Gene (variant)/ CNV** | **Phenotype** | **Gender** | **Parental consanguinity** | **Age (years)** | **Patient** |  |
| Spinocerebellar ataxia (616410) | Homozygous | ***TRPC3*** c.2563C>T p.Leu855Phe (hg38) | Muscle weakness, constipation, hydrocele, headache, kyphosis | M | No | 15 | 20 |  |
|  |  |  |  |  |  |  |  |  |
| KBG syndrome (148050) | Heterozygous | ***ANKRD11*** c.2111_2112delAA p.Lys704llefsTer9, NM_001256182.2 | Hyperbilirubinemia, VSD, dysmorphism, T-cell dysfunction | M | No | 0.4 | 21 |  |
| NA | Hemizygous | ***DUP 265.4kb:chrX:***2811325-3076679 (Hg38) | TGA, dextrocardia, dysmorphism, bilateral ptosis, severe FTT, long eyelashes, facial hemangioma, hepatomegaly, skeletal abnormalities including cranial hyperostosis, femur Erlenmeyer flask deformity, malformations of tibia and fibula | M | Yes | 1.7 | 22 |  |
| NA | Hemizygous | ***TMLHE DEL 2.6kb: chrX:*** 155,511,662-155,514,278, NM_001184797.2 | Headaches, vertigo, anxiety, depression | M | No | 14.4 | 23 |  |
|  | CNV, copy number variation; FTT, Failure to thrive; M, Males; NA: not available; TGA, transposition of the great arteries; VSD, ventricular septal defect | | | | | | |  |
